# Supplementary figures and images for: Self-Reported Long COVID and Its Association with the Presence of SARS-CoV-2 Antibodies in a Danish Cohort up to 12 Months after Infection
Source: Microbiol Spectr. 2022 Nov 9;10(6):e02537-22. doi: 10.1128/spectrum.02537-22 (PMC9769646; doi:10.1128/spectrum.02537-22)

Supplementary figure 1: Serostatus and time since COVID-19 (boxplot)

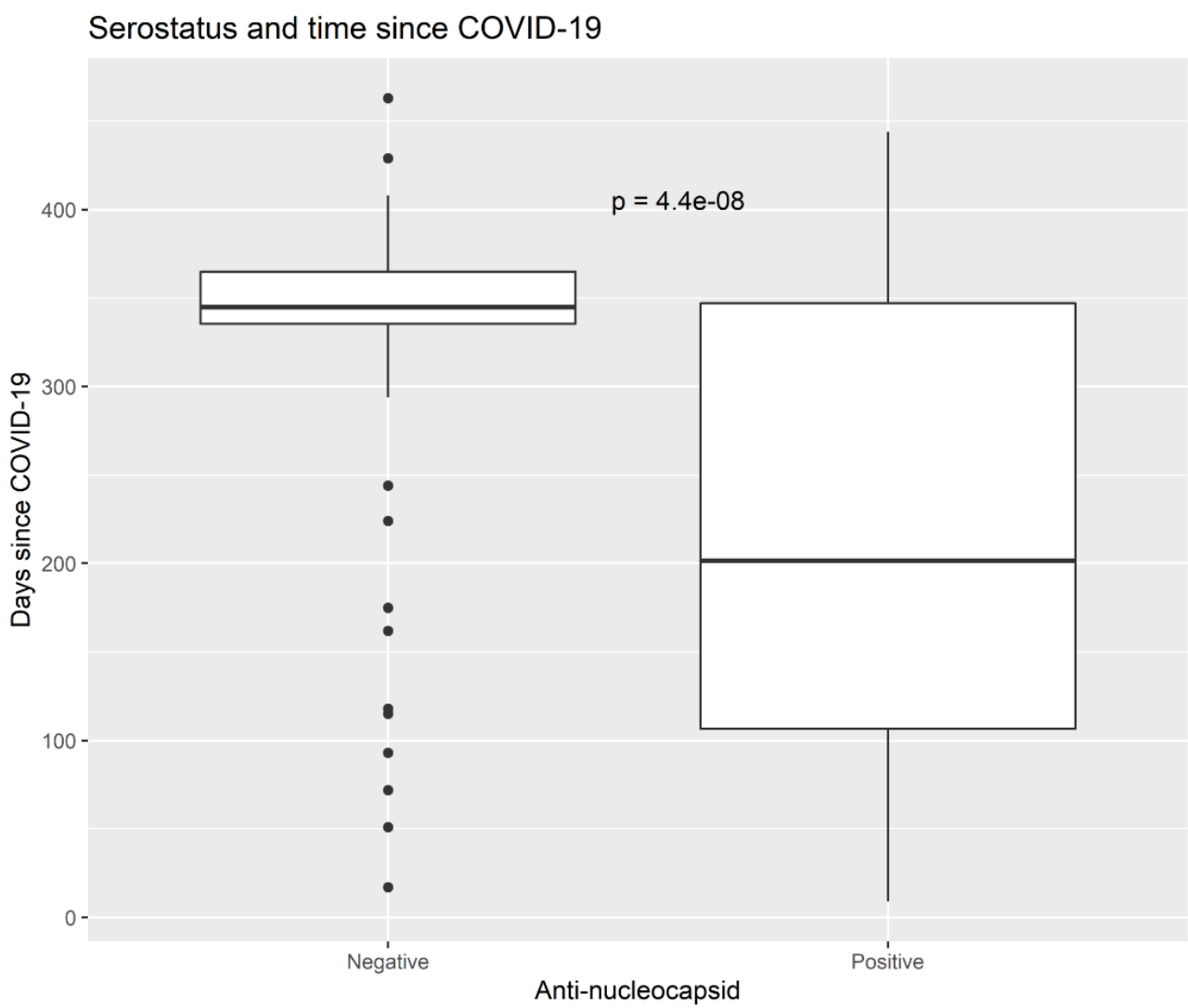

Supplement: Supplemental file 1 — Figure S1. Download spectrum.02537-22-s0001.pdf, PDF file, 0.1 MB [file spectrum.02537-22-s0001.pdf]
